# Aerodynamic and Inertial Loading Effects of Insect-Inspired Appendages in Small Unmanned Aerial Vehicles

**DOI:** 10.3390/biomimetics10010022

**Published:** 2025-01-02

**Authors:** Titilayo Ogunwa, Javaan Chahl

**Affiliations:** 1 UniSA STEM, University of South Australia, Mawson Lakes, SA 5095, Australia; titilayo.ogunwa@unisa.edu.au; 2 Platforms Division, Defence Science and Technology Group, Edinburgh, SA 5111, Australia

**Keywords:** biomimetics, aerodynamic loading, flight dynamics, multibody

## Abstract

Insects enhance aerodynamic flight control using the dynamic movement of their appendages, aiding in balance, stability, and manoeuvrability. Although biologists have observed these behaviours, the phenomena have not been expressed in a unified mathematical flight dynamics framework. For instance, relevant existing models tend to disregard either the aerodynamic or the inertial effects of the appendages of insects, such as the abdomen, based on the assumption that appendage dynamic effects dominate in comparison to aerodynamic effects, or that appendages are stationary. However, appendages in insects exist in various shapes and sizes, which affect the level of both the inertial and aerodynamic contributions to the overall system. Here, the effects of the individual dynamic, inertial and aerodynamic contributions of biologically inspired appendages in fixed wing forward flight demonstrate the utility of the framework on an example system. The analysis demonstrates the effect of these aerodynamic appendages on the steady flight and manoeuvre performance of a small aircraft with an actuated aft appendage capable of movement in the longitudinal and lateral axes, analogous to an insect abdomen. We use the method to consider designs with different appendage areas. The example case showed that ignoring the aerodynamic contribution might yield useful insights depending on the size of the appendage, but including the aerodynamic effects as part of a consistent mathematical framework leads to a more comprehensive understanding of the role of appendage morphology. The method allows improved modelling for modern multivariate control system design using bioinspired appendages. Inertia-dominated appendages provided more advantages in energy-based longitudinal manoeuvres and in trimmed flight, with reduced advantage in initiating lateral manoeuvres.

## 1. Introduction

The reproductive efficiency and survival of many animals rely on their ability to move during activities like migration, territorial defence, foraging, predation, and avoidance [1]. For locomotion in general, controlling body posture is crucial for producing and regulating propulsion forces and the moments needed for control.

Inertial control is found in terrestrial animals; for example, inertial control is seen in actions like twisting during falls, as in rats [2]. Studies on cats have demonstrated their ability to reorient themselves mid-air through complex body rotations, driven by inertial reorientation, which allows them to land on their feet after a fall [3]. Similarly, lizards, such as geckos, use their tails to stabilise and correct body orientation during leaps, which serves as an inertial manoeuvre to maintain balance and control [4,5].

For aerial locomotion, trade-offs between manoeuvrability and stability are crucial in different ecological contexts, affecting how insects move and interact with their environment. Depending on their habitat and ecological needs, insects have evolved various strategies to balance these trade-offs, ensuring robustness in locomotion. For example, insects in complex, cluttered environments may prioritise manoeuvrability to navigate obstacles effectively, while those in open spaces might emphasise stability to maintain steady movement. These strategies reflect the diverse adaptations that have evolved to meet the demands of different ecological niches. Active manoeuvring in flight relies on three main mechanisms: aerodynamic lift and drag, control of body mass distribution, and modifications in moments of inertia [5,6,7,8,9]. Although for aerial locomotion in insects the moments are largely due to the control of the aerodynamics of the wings, an increasing number of studies show that the aerodynamics of body appendages such as the legs and abdomen are also important.

Aerodynamic and inertial effects together are the source of manoeuvrability in flying insects. For example, in hawkmoths, asymmetric wing motions generate significant aerodynamic and inertial torques that are used for steering and maintaining stability [10] (Jankauski et al., 2017). Hawkmoths also utilise both inertial mechanisms and aerodynamic forces for aerial righting. These insects employ abdominal movements and wing adjustments to control their pitch and yaw during flight, illustrating how aerodynamic forces generated by body appendages are used for stability during complex manoeuvres [11]. In addition, investigations into the passive righting mechanisms of dragonflies reveal that their long abdomens and specific wing postures contribute to a passive attitude recovery mechanism during falling [12]. Studies have shown that insects use their legs and abdomen to assist in manoeuvring, with visual and airflow stimuli influencing these movements [13,14,15,16,17,18,19]. Some studies also highlight the importance of leg and abdominal steering in maintaining body posture and manoeuvrability, particularly in insects such as fruit flies and hawkmoths, where these appendages act as aerodynamic rudders aiding in orientation and stability during flight [11,20,21]. Additionally, arboreal ants and other wingless gliding insects use their legs and lateral cerci to control aerial descent, further emphasising the role of aerodynamic forces in manoeuvrability [22,23].

Establishing mathematical models for dynamic systems is important in areas such as control design and performance analysis. The flight dynamics of flapping wing systems are more complex than those of fixed and rotary wing aircraft; however, efforts have been made to develop flight dynamics models of insect-like MAVs for various flight conditions. The common approach in the literature involves using the conventional single-body equations of motion (EoMs) aircraft models, ignoring the effects of moving parts such as the appendages on the entire system. Khan and Agrawal [24] used the standard aircraft model to demonstrate flapping wing MAVs in hover. A quasi-steady aerodynamic model was used to estimate the forces generated by the wings; however, the inertial effects of the wings were neglected. Using averaging theory, a time-averaged longitudinal model of an insect was developed, while neglecting the effects of the inertial and gravitational forces and moments of the wing. The application of averaging theory for insect-inspired flapping wing air vehicles was introduced by Schenato et al. [25,26]. In using this theory, for insects that have high flapping frequency, only the wingbeat-cycle-averaged aerodynamic forces and moments influence the body; therefore, a simplified time-averaging insect model can be developed and used for analysis and control design. Another important consideration is the significant aerodynamic interaction between wing and body, including interference and unsteady effects. Liang and Sun [27] showed maximum interactions of around 5% across aerodynamic force coefficients in a model fruit fly in forward flight and concluded that wing and body effects could be treated separately for the forward flight case for performance purposes. The study herein considers a dragonfly-inspired model, with higher aspect ratio wings, and a narrower body than a fruit fly which should contribute even less to the aerodynamic interaction. Thus, we have treated the wings and body separately for modelling purposes.

For insect-inspired aircraft, most of the multibody flight dynamics models in the literature focus on the degrees of freedom associated with wing motion. Lasek and Sibilski [28] developed one of the first multibody models for flapping wing flight. Gibbs–Appel equations were used to derive the equations of motion for simulation and the degrees of freedom of the wings were limited to two. In Jackson et al. [29], a trajectory optimisation problem was proposed for a flapping wing micro aerial vehicle (FWMAV), modelled as a system of three bodies, where the inertial effects of the wings were included. Bolender used Kane’s equation in [30] to derive the equations of motion for a FWMAV with four rigid bodies. The derivation of the inertial and active forces for the wings was limited and the degrees of freedom of the wings were also limited to two. In [31], Gebert et al. used the Newton–Euler method to derive the multibody EoMs for a FWMAV. The inertial effects of the wings were included; however, simulations were not presented to validate the developed model. To investigate the use of articulated wings for control by controlling the dihedral angle of the wings, Paranjape in [32] developed multibody EoMs using the Newton–Euler method. The model incorporated dynamic centre of gravity variation using the combined centre of mass of the aircraft as the reference point. A tensor-based approach of the Newton–Euler method was used in [33] to derive a simulation model for a FWMAV and only the effects of the wings were considered. In [34], the equations of motion for flapping wing micro aerial vehicles were derived using D’Alembert’s principle. The inertial effects of the wings were included as the developed model was used to analyse the importance of the mass of the wings to flight dynamics stability and control. Hassan and Taha in [35] also used D’Alembert’s principle to derive the multibody flight dynamics equations of motion of FWMAVs, including the wing inertial effects. The equations were then used to analyse the longitudinal flight dynamics of FWMAVs near hover.

Fewer studies have considered the effect or role of appendage movements in flight. A nonlinear model of a dragonfly-like flapping-wing MAV was developed by Du [36]. The study considered the effect of the head and abdomen in developing the equations of motion of a dragonfly-like MAV; however, their movements were not investigated with regards to their effects on the flight, stabilisation or control of the model. Dhyr et al. [17] developed a model to examine the active role of the abdomen in the hawkmoth. However, the model was obtained using a phenomenological, system identification approach. Another study by Tejaswi et al. [37,38] developed a model of the dynamics of a flapping-wing flyer, inspired by monarch butterflies. The model was, however, developed using Euler—Lagrange equations to examine the use of abdomen undulation to improve stability and reduce energy and power consumption.

A review of the literature showed that existing flight dynamic models of insects or insect-inspired air vehicles mostly ignore the aerodynamic and/or inertial effects of appendages. Studies that have ignored the aerodynamic effect of the appendage, such as the abdomen for instance, have done so based on the assumption that the inertial effects of the abdomen are more significant than the aerodynamic effects and that these aerodynamic effects are less substantial compared to those of the head, thorax, and wings combined. However, the range of body sizes and shapes of flying insects is quite diverse, with body lengths spanning six orders of magnitude. For instance, the body length of fairy wasps (*Dicopomorpha echmepterygis*) is approximately 0.13 mm [39], while that of extinct massive protodonates could have been up to 350 mm [40]. There is also a considerable amount of variation in the body shape of flying insects, just as there is in body size. For example, dragonfly and damselfly abdomens are elongated, while flying beetle bodies are stout. The mechanisms of appendage movement in flying systems can be classified based on the operation by way of inertia and operation based on aerodynamic torque [3]. Depending on the mass, size and shape of the appendage, the contributions of either of these mechanisms to the overall system vary; hence, the significance of these contributions comes into question.

In previous studies [41,42], we developed a multibody model for a dragonfly-inspired straight-wing unmanned aerial vehicle (UAV) model with a deflectable abdomen; however, our analyses only included the inertial effects of the abdomen. Here, we extend this work by including the aerodynamic contribution of the abdomen to investigate its effect on steady cruise flight and pull up manoeuvre performance. The effectiveness associated with abdominal deflection for manoeuvring is also evaluated: specifically, how some physical and kinematic properties of the abdomen affect the manoeuvre effectiveness. Three distinct models used throughout this paper are defined as follows: (1) the simplified or “inertial” model, which includes only the dynamic/inertial effects of the deflectable abdomen; (2) a modified or “inertial + aero” model, which includes the inertial and aerodynamic effects of the deflectable abdomen; and (3) the modified model with twice the nominal abdominal area, which is equivalent to increasing the effective area (aerodynamic contribution) of the abdomen without a weight penalty.

The contribution of this study is to establish a mathematical framework to allow aircraft designs inspired by insect flight controls to integrate simultaneous inertial and aerodynamic strategies to achieve precise control during complex aerial manoeuvres. The included example case demonstrates how combining inertial and aerodynamic forces through appendage and wing configuration enables conceptual novel platform design, and control system architectures for biomimetic aircraft with articulated appendages.

Throughout this paper, we will use terms from insect anatomy to describe the analogous components of conceptual aircraft used for modelling. For example, the articulated tail will be referred to as an abdomen, since a normal aircraft tail assembly does not have a large multi-axis articulated joint.

The remainder of this paper is as follows: Section 2 introduces the multibody equations of motion. Section 3 derives the aerodynamic effects of the abdomen. Section 4 gives the specification of the aircraft model. Section 5 demonstrates the effect of the aerodynamics of the abdomen on aircraft flight performance. Section 6 demonstrates the effect of abdominal deflection on manoeuvring. Section 7 details the outcomes of this paper, and Section 8 concludes the paper.

## 2. Multibody Equations of Motion

**Figure 1:**
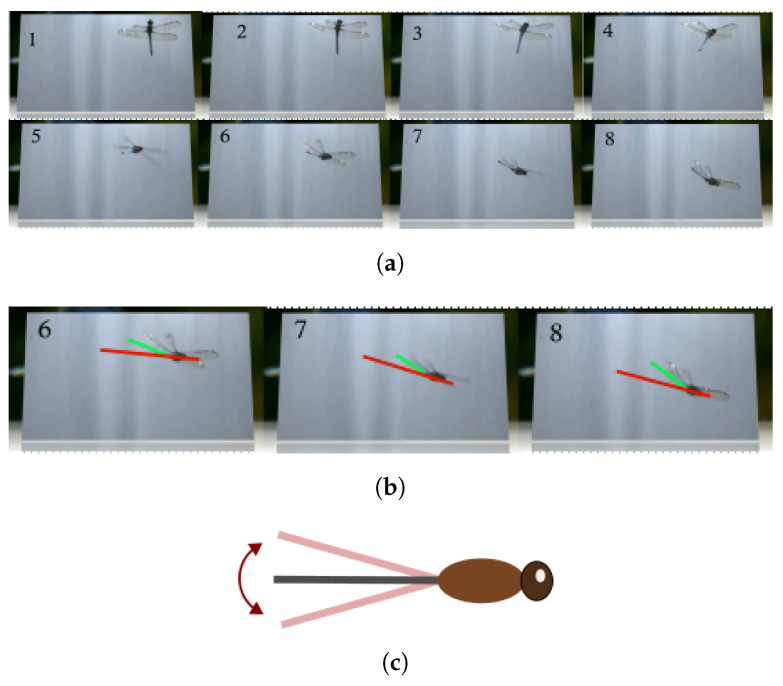
Dragonfly in diving manoeuvre (video taken from [43]). (**a**) A dragonfly performing a longitudinal manoeuvre. The frame rate in these is 24 fps and every 10th frame is shown. (**b**) Specific frames that show the motion of the abdomen. The green line represents the centreline of the abdomen and the red line represents the centreline of the body. (**c**) Simplified representation of the abdominal motion (side view).

**Figure 2:**
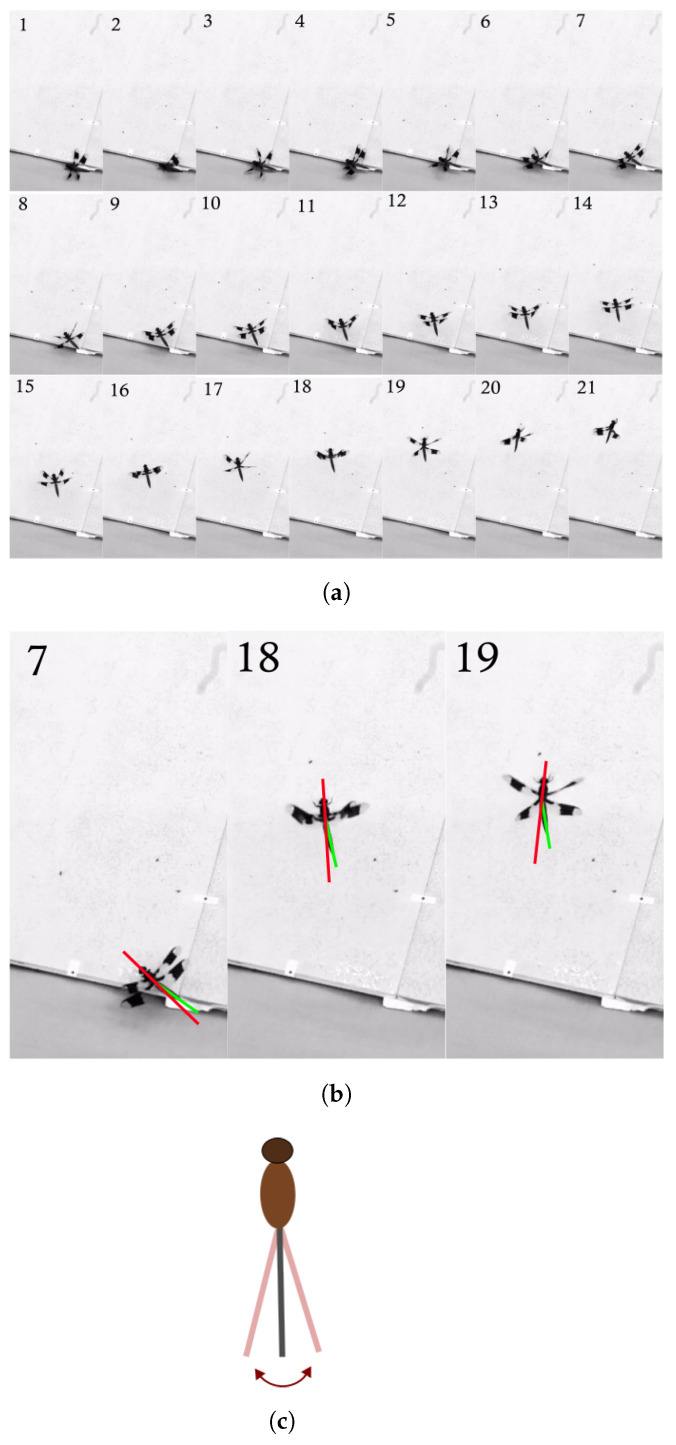
Dragonfly (*Plathemis lydia*) in turning flight for prey capture (video taken from [44]): (**a**) A dragonfly performing a lateral manoeuvre. The frame rate in these is 28 fps and every 10th frame is shown. (**b**) Specific frames with abdominal movements. Green line represents the centreline of the abdomen and the red line represents the centreline of the body. (**c**) Simplified representation of the abdominal motion (top view).

To develop equations of motion that reflect the effect of abdominal movement, a series of forward flight dragonfly manoeuvres were observed and are shown in Figure 1 and Figure 2. Note the nature of abdominal motion in flight by the deflection of the abdomen relative to the body about the pitch axis in Figure 1, and yaw axis in Figure 2. Throughout this framework development, when referring to biological data for dragonflies, we have gleaned observations from multiple studies in the literature when assessing flight and performance. Different species are likely to have different abdominal articulation, dynamic characteristics and kinematic options due to different morphologies.

**Figure 3:**
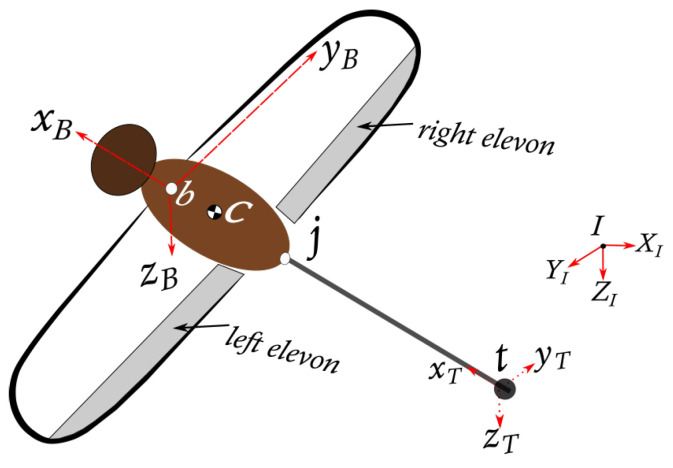
Reference points and coordinate systems of the dragonfly-inspired straight-wing aircraft.

The aircraft (Figure 3) considered for this study includes an articulated abdomen appendage analogue. The fore and hind wings are combined to form single wings with elevons; thus, the equations of motion are simplified to a two-body problem. Considering other analogies to a dragonflythe head/thorax/wings combination is abstracted as a rigid body *B* with a mass $m^{B}$ and will be referred to as the *central body*. The central body typically has six degrees of freedom: three translational and three rotational. The abdomen or *tail* as the second rigid body, *T*, is modelled as an abdominal mass $m^{T}$ with three rotational degrees of freedom. The whole aircraft (*central body + tail*) with mass *m* will be referred to as the rigid body *C*. Four reference points (b,c,t and *j*) are used to represent the centres of mass of the body, whole aircraft, tail, and tail joint, respectively. The reference frames $\left(X_{I},Y_{I},Z_{I}\right)$, $\left(x_{B},y_{B},z_{B}\right)$ and $\left(x_{T},y_{T},z_{T}\right)$ represent the inertial, central body and tail reference frames, respectively.

This paper considers an aircraft with analogies to dragonfly anatomy, which is dominated by wings and abdomen. The two-body model provides useful insights; however, the models can easily be extended for a system with more than two rigid bodies. The dynamic equations are referenced to point *b* and written in the central body fixed reference frame *B*.

The translational dynamic equation of motion for the aircraft model as derived in our previous work [42] is as follows:

(1) $$\begin{array}{cc} \; & m\;\left({\left[{\dot{V}}_{B}^{I}\right]}^{B}+{\left[\Omega^{BI}\right]}^{B}{\left[V_{b}^{I}\right]}^{B}\right)+m^{T}({\left[R\right]}^{BT}({\left[{\dot{\Omega }}^{TB}\right]}^{T}{\left[s_{tj}\right]}^{T}+{\left[\Omega^{TB}\right]}^{T}{\left[\Omega^{TB}\right]}^{T}{\left[s_{tj}\right]}^{T})\\ \; & +{\left[{\dot{\Omega }}^{BI}\right]}^{B}\left({\left[R\right]}^{BT}{\left[s_{tj}\right]}^{T}+{\left[s_{jb}\right]}^{B}\right)+\left({\left[\Omega^{BI}\right]}^{B}\;{\left[\Omega^{BI}\right]}^{B}\left({\left[R\right]}^{BT}{\left[s_{tj}\right]}^{T}+{\left[s_{jb}\right]}^{B}\right)\right)\\ \; & +\left(2\;\Omega^{BI}{\left[R\right]}^{BT}\left({\left[\Omega^{TB}\right]}^{T}{\left[s_{tj}\right]}^{T}\right)\right))=F_{B}+F_{T} \end{array}$$

where $s_{tj}$ is the displacement vector of the abdomen centre of mass relative to the joint. $V_{b}^{I}$ and ${\dot{V}}_{B}^{I}$ are the translational velocity and acceleration of the central body with respect to (wrt) the inertial frame, respectively. $\Omega^{BI}$ and ${\dot{\Omega }}^{BI}$ are the the skew symmetric matrix of the angular velocity and acceleration of the central body mass wrt the inertial frame, respectively. $\Omega^{TB}$ and ${\dot{\Omega }}^{TB}$ are the skew symmetric matrix of the angular velocity and acceleration of the tail mass wrt the body frame, respectively. ${\left[R\right]}^{BT}$ is the transformation matrix from abdomen to body coordinate system. $F_{B}$ and $F_{T}$ represent the contributing sum of all the forces acting on the aircraft from the central body and tail, respectively.

The rotational dynamic EoM for the aircraft is

(2) $$\begin{array}{cc} \; & m^{T}\left({\left[R\right]}^{BT}{\left[S_{tj}\right]}^{T}+{\left[S_{jb}\right]}^{B}\right)({\left[R\right]}^{BT}\left({\left[{\dot{\Omega }}^{TB}\right]}^{T}{\left[s_{tj}\right]}^{T}+{\left[\Omega^{TB}\right]}^{T}{\left[\Omega^{TB}\right]}^{T}{\left[s_{tj}\right]}^{T}\right)\\ \; & +{\left[{\dot{\Omega }}^{BI}\right]}^{B}\left({\left[R\right]}^{BT}{\left[s_{tj}\right]}^{T}+{\left[s_{jb}\right]}^{B}\right)+\left({\left[\Omega^{BI}\right]}^{B}\;{\left[\Omega^{BI}\right]}^{B}\left({\left[R\right]}^{BT}{\left[s_{tj}\right]}^{T}+{\left[s_{jb}\right]}^{B}\right)\right)\\ \; & +\left(2\;\Omega^{BI}{\left[R\right]}^{BT}\left({\left[\Omega^{TB}\right]}^{T}{\left[s_{tj}\right]}^{T}\right)\right)+\left({\left[{\dot{V}}_{B}^{I}\right]}^{B}+{\left[\Omega^{BI}\right]}^{B}{\left[V_{b}^{I}\right]}^{B}\right))\\ \; & +{\left[I_{b}^{B}\right]}^{B}{\left[{\dot{\omega }}^{BI}\right]}^{B}+{\left[\Omega^{BI}\right]}^{B}{\left[I_{b}^{B}\right]}^{B}{\left[\omega^{BI}\right]}^{B}={\left[M_{B+T}\right]}^{B}+\left[{\left[S_{tb}\right]}^{B}{\left[F_{T}\right]}^{B}\right] \end{array}$$

where $S_{tj}$ is the skew symmetric matrix of the displacement vector of the abdomen centre of mass relative to the joint. $s_{jb}$ and $S_{jb}$ are the displacement vector and skew symmetric matrix of the displacement vector of the joint relative to the central body’s centre of mass. $I_{b}^{B}$ is the inertia tensor of rigid body *B* about point *b*. $M_{B+T}$ represents the contributing sum of all the moments acting on the aircraft from the central body and the tail.

## 3. Aerodynamic Loading of the Abdomen

**Figure 4:**
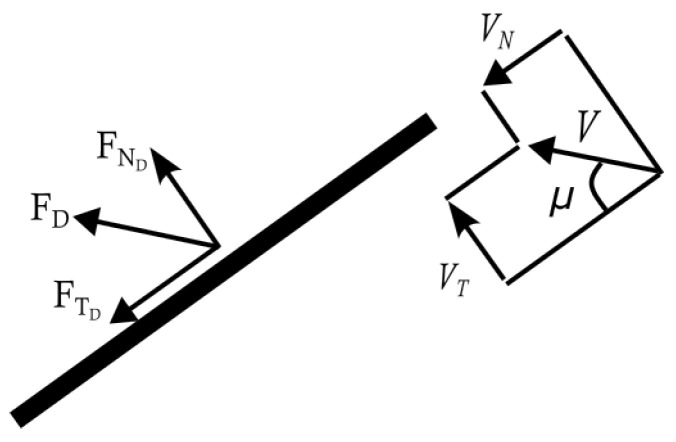
Inclined cylindrical bar in a flow.

The forces and moments acting on the aircraft are due to aerodynamics, gravity and propulsion, and have been detailed in our previous studies [41,42]; therefore, only the aerodynamic effects of the abdomen are discussed in this section, as that is the focus of this study. The equations presented in Section 2 assume that the aerodynamic contributions of the dragonfly’s body and tail are independent and additive. The elongated abdomen of the dragonfly and the UAV considered in this study are comparable to thin long cylinders. It is also assumed that, compared to the drag, the lift force produced by the abdomen is negligible [45]. The drag force ($F_{D}$) of a cylinder inclined at an angle μ to the airflow, *V*, can be decomposed into the normal $F_{N_{D}}$ and tangential $F_{T_{D}}$ directions, as shown in Figure 4.

These forces can be derived from Equation (3) [45]:

(3) $$F_{N_{D}}=\frac{C_{N}}{2}\rho V_{N}^{2}S_{ref},\;F_{T_{D}}=\frac{C_{T}}{2}\rho V_{T}^{2}S_{ref}$$

where $V_{N}$ and $V_{T}$ are the normal and tangential component of the velocity *V*, respectively, and ρ is the density of air. The $S_{ref}$ used is the projected plan area of the body, equal to the product of body diameter, $\left(d_{t}\right)$, and length $\left(l_{t}\right)$.

Therefore, the aerodynamic drag is estimated in terms of normal and tangential forces. The resolved coefficients $C_{N}$ and $C_{T}$ both vary with Reynolds number (Re) and the global approximation model of these coefficients is given by Ellington [45]. The estimation of Re is based on the length of the abdomen. The normal drag coefficient $C_{N}^{\prime }$ of very (infinitely) long cylinders is given by [45]

(4) $$C_{N}^{\prime }=1.1+22/Re$$

where the *Re* is based on the length of the cylinder, $l_{t}$. For cylinders with finite length, the normal drag coefficient is given by [45]:

(5) $$C_{N}/C_{N}^{\prime }=0.57+0.34e^{-7.6d_{t}/l_{t}}$$

**Figure 5:**
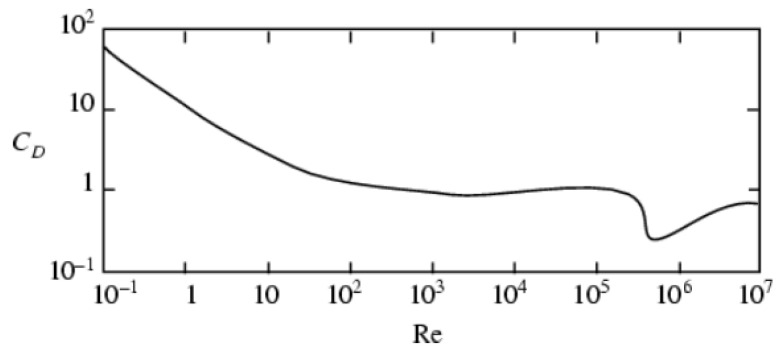
Variation in cylinder drag coefficient with Reynolds number [46].

The results using the conceptual UAV with $\left(l_{t}/d_{t}=8\right)$ for local airspeeds ranging from 5 to 15 m/s result in $Re=10^{5}$. Figure 5 shows the variation in drag coefficient as a function of Re for a circular cylinder [46]. $C_{D}$ decreases monotonically from high values for low Re to near one at Re ≈ 300,000. At this point, the $C_{D}$ suddenly drops from around 1 to around 0.3, a phenomenon known as the drag crisis. After that, $C_{D}$ returns to around 0.6 for Re = $10^{7}$.

In this study, it is assumed that the drag coefficient across the range of Reynolds numbers tested is fairly constant and is solely dependent on the inclination angle relative to the airflow (see Figure 4). For an inclined cylinder, the effect of the inclination angle can be represented by revising Equation (3) as [47]:

(6) $$F_{N_{D}}=\frac{C_{N}}{2}\rho S_{ref}V^{2}sin^{2}\left(\mu \right),\;F_{T_{D}}=\frac{C_{T}}{2}\rho S_{ref}V^{2}cos^{2}\left(\mu \right)$$

However, research has shown that the classic decomposition in Equation (6) does not match experimental data very well, particularly for small angles of attack [47]. Hence, the load function method is a more practical approach, in which [47]

(7) $$F_{N_{D}}=0.5\rho S_{ref}V_{N}^{2}f_{N}\left(\mu \right),\;F_{T_{D}}=0.5\rho S_{ref}V_{T}^{2}f_{T}\left(\mu \right)$$

Lift and drag coefficients ($C_{L}$ and $C_{D}$) are related to the normal and tangential coefficients by [48]

(8) $$\left[\begin{array}{c} C_{L}\\ C_{D} \end{array}\right]=\left[\begin{array}{cc} c\;\mu & -s\;\mu \\ s\;\mu & c\mu \end{array}\right]\left[\begin{array}{c} C_{N}\\ C_{T} \end{array}\right]$$

Therefore, considering the lift and drag coefficients of a cylinder originally given by Hoerner [49], the approximation of load functions $f_{N}\left(\mu \right)$ and $f_{T}\left(\mu \right)$ in [47] are used:

(9) $$\begin{array}{cc} f_{N}\left(\mu \right) & =0.02\sin\left(\mu \right)+1.1\sin^{4}\left(\mu \right)+1.1\sin^{2}\left(\mu \right)\cos^{2}\left(\mu \right) \end{array}$$

(10) $$\begin{array}{cc} f_{T}\left(\mu \right) & =0.02\cos(\mu ) \end{array}$$

**Figure 6:**
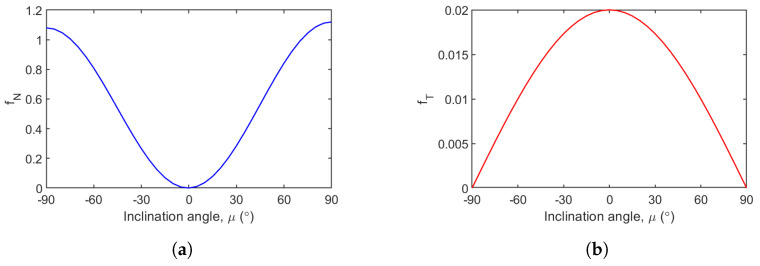
Variation in drag load functions with inclination angle. (**a**) Variation in normal drag load function with inclination angle. (**b**) Variation in tangential drag load function with inclination angle.

The resulting plots showing the variation in load functions with inclination angle are shown in Figure 6. Note that the angle is in degrees.

The aerodynamic torque due to the resulting drag of the abdomen is estimated by ${\left[s_{tb}\right]}^{B}\times {\left[F_{D}\right]}^{B}$. Using the abdominal pitch angle ($\theta_{T}$) and pitch angle of the central body (θ), the inclination angle between the airflow and the local longitudinal axis is given by the abdomen effective angle of attack $\alpha_{T}$ and is obtained as $\alpha_{T}=\theta +\theta_{T}-\mathrm{atan}\frac{{\left[V_{T_{z}}^{I}\right]}^{T}}{{\left[V_{T_{x}}^{I}\right]}^{T}}$. Because of the symmetry of the cylinder, the same estimation will be used for the inclination angle between the airflow and the local lateral axis: the effective sideslip angle $\beta_{T}$, which is obtained similarly to the abdomen effective angle of attack.

## 4. Aircraft Specification

The aircraft model from [41], with the properties summarised in Table 1, was used for the simulation. The aerodynamic properties of the aircraft’s central body were assumed to be solely produced by the wings and were obtained using the open source tools, Athena Vortex Lattice (AVL) and XFLR5 [50,51,52]. AVL, developed by Drela, uses an extended vortex–lattice method for lifting surfaces to generate aerodynamic derivatives data. AVL produces relatively accurate aerodynamic data for conceptual and preliminary design stages, and is widely used both in industry and academia [53,54]. The tool takes an input file containing the aircraft geometric parameters defined in sections, and computes the static stability, dynamic stability, and control derivatives. See Appendix A for the AVL geometry file used in this study. More information on how to use the AVL tool can be found in [50].

The aerodynamic derivatives were generated for a range of angles of attack and sideslip angles, and implemented in the form of lookup tables, which were then used to find the corresponding force and moment coefficients for aircraft states. Outside of the data envelope, the values for the simulation were either set to the value of the last data point or extrapolated.

One limitation of AVL is that it does not capture viscous effects and can only predict lift-induced drag. To account for viscous effects, a constant pressure drag was estimated using XFLR5 and included in the AVL input geometry file. These derivatives were calculated in the body-fixed frame and follow conventionally accepted standards with regard to nomenclature and non-dimensionality.

XFLR5 extends the 2D solutions of XFOIL to 3D applications using analysis tools such as the vortex–lattice method, nonlinear lifting line theory and 3D panels. Viscous calculations can also be interpolated from XFOIL data [55]. XFLR5 software provides a graphical user interface for aircraft design, with mass and inertia estimation capabilities using component point masses. More information on how to use XFLR5 can be found in [52,56].

**Figure 7:**
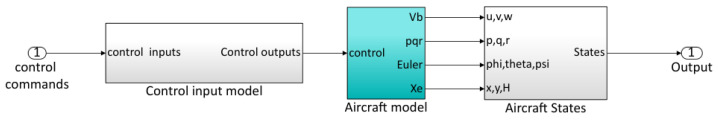
Aircraft model in Simulink.

The implementation of the mathematical models was carried out in the MATLAB/Simulink simulation environment. Figure 7 shows the Simulink model of the aircraft. The “control input model” produces the control inputs, which include thrust $\left(T_{n}\right)$ for propulsion; aerodynamic control surfaces, which are the left and right elevons that function as elevators ($\delta_{e}$) or ailerons ($\delta_{a}$); and the tail roll $\left(\phi_{T}\right)$, pitch $\left(\theta_{T}\right)$ and yaw $\left(\psi_{T}\right)$ angular deflections relative to the body frame. Where applicable, a downward deflection of a control surface or tail deflection is positive and an upward deflection is negative.

The “Aircraft model” contains the nonlinear equations of motion, including the external forces and moments due to aerodynamics, propulsion and gravity, which then produce the aircraft state vector (u,v,w,p,q,r,ϕ,θ,ψ,x,y,H). Also, an initial verification/validation of the proposed multibody model is presented in Appendix B.

## 5. Performance Effects of Appendages

This section demonstrates the effects abdominal movements have on various areas of forward flight performance in simulation. Steady cruise and turning flight performances are examined. The performance parameters that define the vehicle’s steady performance characteristics for the UAV were selected given that it is not a question of whether the abdomen exists or is in use or not, but a question of what effects it has on performance, given that it is already present and is capable of different motions.

### 5.1. Steady Cruise Flight

As mentioned earlier, insect abdomens are found in different shapes and sizes, both factors that contribute to the aerodynamic and inertial effects of the abdomen. The performance analysis carried out in this section focused on steady cruise, where the effect of including the abdominal aerodynamic loading and increasing the abdominal area at various cruise speeds was investigated. The abdominal pitch angle was varied (from −60° to 60°) while trimming the aircraft using the elevator for three cruise speeds, 5, 10 and 15 m/s, at a height of $\left(H_{0}=100\;m\right)$. The analysis compared results from the simplified model, the modified model, and the modified model with twice the nominal abdominal area. The performance metrics used were the power required and the control effort required from the elevator at various abdominal pitch angles.

During steady cruise, the lift generated by the aircraft equals its weight (L=W) and the thrust produced balances the drag $\left(T_{n}=D\right)$, resulting in zero specific power. The energy required during cruise $E_{req}$ over time can be calculated by integrating the power required $P_{req}$ over the flight duration:

(11) $$E_{req}=\int_{t}^{tf}P_{req}dt,$$

**Figure 8:**
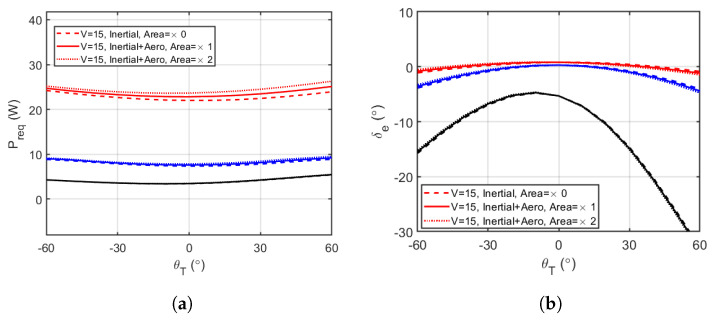
Analysis of elevator trimmed-level flight for a range of tail deflections, with mass (inertial) effects and two tail geometries with different aerodynamic properties due to different surface areas. The black, blue and red lines represent results for V = 5, 10 and 15 m/s, respectively. (**a**) Power required vs. abdominal pitch angle. (**b**) Elevator deflection vs. abdominal pitch angle. For this configuration, the power required is slightly affected by the deflection of the tail after the addition of elevator trim. The balance effects of the angle of the tail are most pronounced in causing a need for trim input from the elevator, particularly at low air speed.

In Equation (11) above, $P_{req}$ represents the power needed per second during cruise, which is equal to the product of drag and velocity $\left(P_{req}=DV\right)$. This analysis assumes that the aircraft always has enough thrust to overcome the drag. The results for the power required and elevator deflection required to trim the aircraft for steady cruise flight at varying abdominal deflections are shown in Figure 8a and Figure 8b, respectively. The percentage average variation in steady cruise flight performance parameters compared to the simplified/inertial model is also summarised in Table 2.

### 5.2. Quasi-Steady Pull Up Manoeuvre

The open loop fixed-thrust pull manoeuvre performance is analysed in this section. The manoeuvrability advantage in utilising either the elevator or abdominal deflection to initiate a manoeuvre from trimmed cruise flight was examined, using the simplified and modified models. The same energy-based performance metrics (specific energy and specific excess power) were used for comparison.

**Figure 9:**
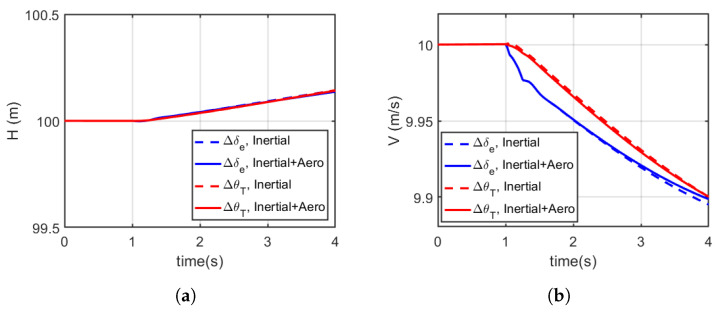
Pull up manoeuvre time response considering, in blue, elevator-induced manoeuvre and, in red, inertia tail deflection, with and without aerodynamic effects. (**a**) Height (H). (**b**) Airspeed (V). It is apparent that there is an instantaneous advantage in preserved velocity when using the appendage to initiate the manoeuvre in this aircraft, with apparently slightly more advantage for a non-aerodynamic tail.

**Figure 10:**
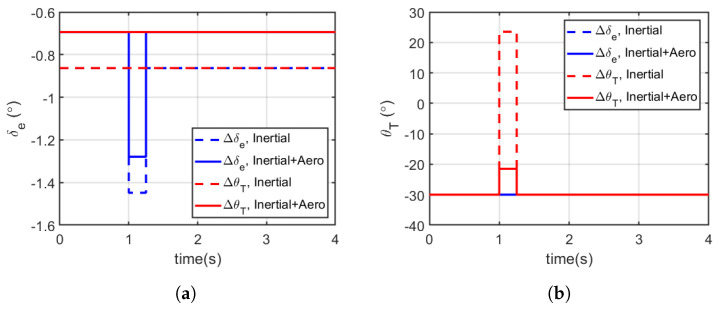
Pull up manoeuvre and longitudinal control input time response. These plots illustrate the control effort required to execute the pull up manoeuvre, comparing the performance of different models. (**a**) Elevator deflection ($\delta_{e}$). (**b**) Abdominal pitch angle ($\theta_{T}$). To achieve the same effect, the inertia only tail moves substantially in angle compared to the inertial/aerodynamic tail.

**Figure 11:**
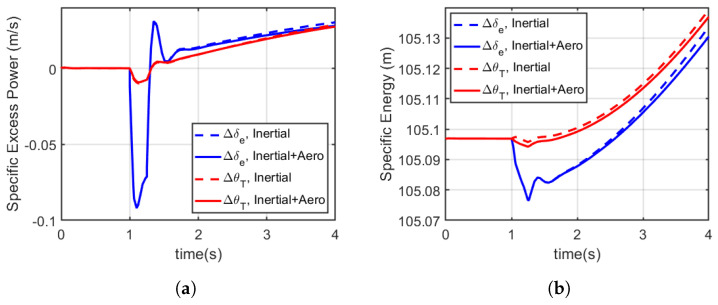
Manoeuvre advantage measured as specific excess power and specific energy at 10 m/s. These plots compare how the inertially manoeuvring aircraft (red) vs. the elevator manoeuvring aircraft (blue) respond energetically during the pull up manoeuvre. (**a**) Specific excess power. (**b**) Specific energy. The inertia only tail provides a slightly higher instantaneous energy advantage over the inertial and aerodynamic tail, and the inertial effect provides an energy advantage of about 15 mm over the elevator-based manoeuvre about 0.25 s after the onset of the manoeuvre.

For a duration of 4 s, the pull up manoeuvre was initiated using a pulsed input signal (applied at t=+1 in the simulation for 0.25 s). The time history for airspeed and trajectory is shown in Figure 9, while the corresponding elevator deflection and abdominal pitch control inputs are shown in Figure 10, and specific excess power and specific energy as a function of time are shown in Figure 11. Table 3 summarises the average specific energy and power over 4 s, using the various models and control effector combinations.

### 5.3. Discussion of Performance Effects of Appendage

In analysing the effect of the inclusion of the abdominal aerodynamic load has on longitudinal performance, Figure 8a shows the expected result that the power required increased with increasing airspeed, while Figure 8b showed that the absolute values of required elevator deflection angle decreased with increasing airspeed, exactly as they would for a control surface. From the summary presented in Table 2, including the aerodynamic loading on the abdomen, we increased the average power required, which was also an obvious effect. In addition, the power required increased with increasing cruise speed, with the least increase of 0.77% recorded for V = 5 m/s, and the highest increase of 3.64% recorded for V = 15 m/s. The overall increase in power required was due to the introduced additional drag from the abdomen increasing the total drag, and hence the power and energy required consequentially. The elevator deflection angle initially increased in average value by 0.17% at V = 5 m/s but decreased by an average of 3.20% at V = 15 m/s. The most significant observation from these results is that a cylinder nominally parallel to the free stream is a poor configuration for an elevator-like control surface.

Thus, increasing the abdominal area further increases the power required. Compared to the nominal abdominal area, an increase in area by a factor of 2 caused the average power required to increase by a minimum of 1.53% at V = 5 m/s, and a maximum of 7.28% at V = 15 m/s. Therefore, aerodynamic effects become more significant as the area of the abdomen increases, especially at higher speed. This is because the drag force is directly proportional to the area; hence, increasing the area of the abdomen increases the total drag force, which causes an increase in power required in steady cruise flight. With twice the abdominal area, the elevator deflection angle initially increased in average value by 0.33% at V = 5 m/s but decreased by an average of 6.97% at V = 15 m/s. In addition, from Figure 8b, it can be seen that the least cruise speed of 5 m/s required the highest absolute elevator deflection angles to trim, and the results for that particular cruise speed were only valid for abdominal pitch angles between $-60^{\circ }$ and $40^{\circ }$, where the lower limit of the elevator deflection angle of $-20^{\circ }$ was exceeded. These observations begin to indicate that the movement of the abdomen may be a more significant factor at low speeds, where its balance effects are high compared to available aerodynamic authority.

Figure 9a,b show how the aircraft’s altitude and speed change over time as it executes the pull up manoeuvre. The data show that the aircraft experiences a noticeable increase in height while undergoing a corresponding decrease in airspeed, reflecting the trade-off between altitude gain and speed loss typical in such manoeuvres. The results also compare different models, showing variations in the response depending on whether aerodynamic and inertial effects are considered.

The specified pull up manoeuvre trajectory (Figure 9a) was achievable using the various models and control effector combinations considered. However, with the inclusion of aerodynamic loading on the abdomen, the modified models required less control effort from the abdomen and elevator to initiate the manoeuvre from trim compared to the simplified models (see Figure 10a,b). It is worth noting that the abdomen was initialised and already deflected and so reducing the deflection angle reduced the overall drag and the control effort required in return. Figure 11a,b show the show the specific excess power and the specific energy as a function of time, which is summarised in Table 3. From the summary presented in Table 3, with the modified model, the average specific excess power and energy reduced whether using the elevator or abdomen deflection, albeit by a minute amount (less than 0.001% on average). In addition, even with the inclusion of the aerodynamic loading of the abdomen, using the abdomen deflection still maintained a higher manoeuvre advantage as the average specific energy and excess power were higher than the aerosurface alternative.

Overall, the results from the performance analyses indicate that the inclusion of the aerodynamic effects of the abdomen reduce the performance of steady cruise in terms of increased power required and also quasi-steady pull up manoeuvre in terms of reduced average specific energy and excess power, indicative of lower manoeuvre advantage. Hence, an inertial dominant appendage might be more beneficial than an aerodynamically dominant appendage or abdomen as an alternative control effector in flight, particularly when survivability in aerial combat is a primary concern.

## 6. Manoeuvring Using Abdominal Deflection

**Figure 12:**
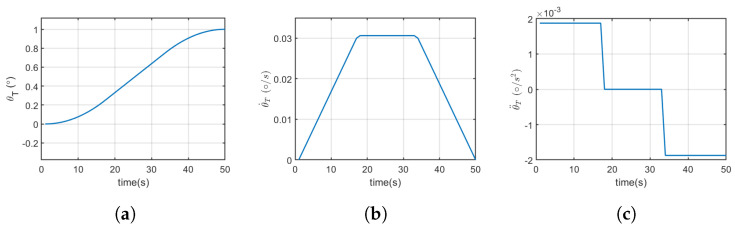
Linear segment with parabolic blend (LSPB). (**a**) Angular deflection. (**b**) Angular velocity. (**c**) Angular acceleration.

Manoeuvrability is essential for survival in the insect world. Throughout missions, an aircraft’s manoeuvre is one of the critical factors that determines its outcome. As opposed to steady flight conditions, manoeuvrability has to do with transient responses; hence, acceleration is an important component of manoeuvrability. Therefore, in flight, manoeuvrability can simply be described as the ability to change the magnitude of the velocity vector (acceleration or deceleration) and/or change in its attitude. For our investigation in this section, manoeuvrability is strictly related to enabling dynamic behaviours or initiating manoeuvres with the deflection of the abdomen from trimmed cruise flight with fixed thrust. It measures the open loop capabilities of the aircraft; hence, the objective is quickness and the quicker the better. To ensure consistency in the abdominal deflection motion, we defined a Linear Segment with Parabolic Blend (LSPD) for the trajectory, a simple trapezoidal profile for velocity and polynomial segments for acceleration and deceleration. The *lspd* function in the Robotics toolbox [57] in MATLAB was used to generate the LSPD trajectories such that it chose the velocity of the linear segment. Figure 12 below shows an example of a $0^{\circ }$ to $1^{\circ }$ deflection, with the velocity and acceleration profile generated using the *lspd* function. In the sections that follow, the effects of the aerodynamic load of the abdomen, increased abdominal area and different abdominal angular velocities on effectiveness are analysed in both longitudinal and lateral axes.

### 6.1. Effect of Increased Abdominal Area on Longitudinal Manoeuvring

This section uses comparative analysis to demonstrate the effects of including the aerodynamic loading of the abdomen and increasing the abdominal area on the effectiveness for longitudinal manoeuvring due to the deflection of the abdomen along the longitudinal axis (as in Figure 1). The metric used to quantify effectiveness is the maximum achievable rate of change in velocity and/or attitude over a specific time period. The longitudinal aerosurface (elevator) was not used for initiating a manoeuvre from trim at any point.

**Figure 13:**
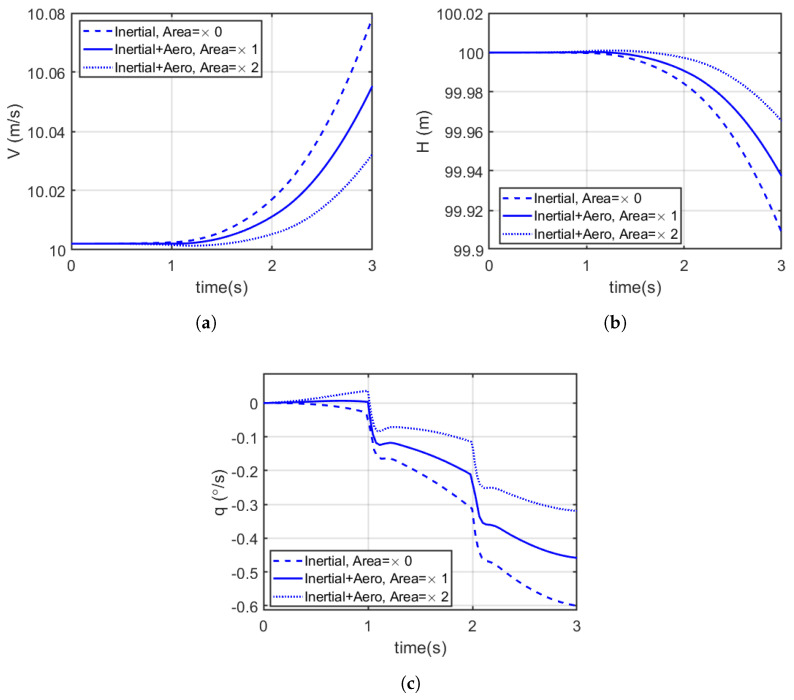
Time history of longitudinal states during the defined manoeuvre initiated by abdominal deflection for an appendage that is only inertial, one that has aerodynamics, and one that has aerodynamics with twice the surface area, all in the dragonfly-inspired aircraft model: (**a**) Velocity (V). (**b**) Height (H). (**c**) Pitch rate (q). Increasing surface area increases drag, which is apparent in the decreased acceleration resulting from the pitch down and decreased change in rate of descent compared to the inertial-only case. Pitch rate is most rapid for the inertia-only appendage case, and decreases with increasing appendage surface area.

The aircraft was trimmed to a steady cruise flight of V = 10 m/s and a height of H = 100 m. Then, the abdomen was deflected upwards from 0 to −10° using a pre-defined velocity profile. The analysis compared three models: the simplified model, the modified model and the modified model with twice (2×) the nominal abdominal area. Only the first 3 s after the initiation of the manoeuvre with the abdomen are presented below in Figure 13, while Table 4 shows the percentage average variations compared to the simplified model with nominal abdominal area.

### 6.2. Effect of Increased Abdominal Area on Lateral Manoeuvring

This section uses comparative analysis to test the the effect of abdominal area on lateral manoeuvring effectiveness due to the deflection of the abdomen along the lateral axis (as in Figure 2). The metric used to quantify effectiveness is the maximum achievable rate of change in velocity and/or attitude over a specific time period. The aerosurfaces (left and right elevons) were not at any point used to initiate the manoeuvre from trim.

**Figure 14:**
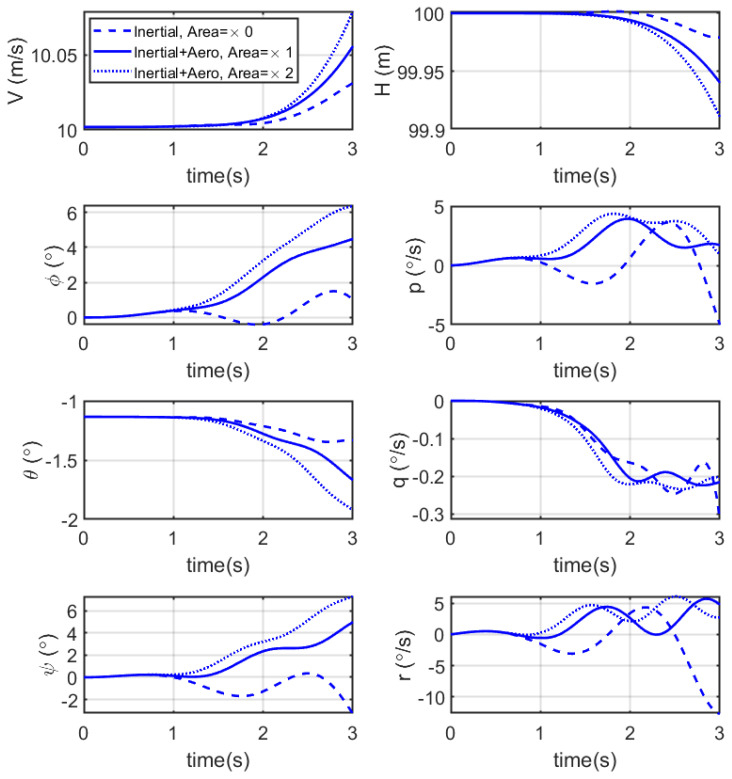
Time response comparison of the lateral–directional states during a manoeuvre initiated by lateral abdominal deflection: velocity (V) and height (H), roll angle (ϕ) and roll rate (p), pitch angle (θ) and pitch rate (q), yaw angle (ψ) and yaw rate (r). The inertia-only appendage was the least effective in achieving yaw, while also preserving the most energy, due to less parasitic drag and less induced drag, with effectiveness increasing for appendages with more surface area. The shift in centre of gravity destabilises the aircraft, which is most apparent with the absence of aerodynamic damping for the inertia-only appendage; this variable stability effect can potentially be exploited by a sophisticated controller.

The aircraft was first trimmed to a steady cruise flight of V = 10 m/s and height H = 100 m. Then, the abdomen was deflected rightward from 0° to −5° at the same rate. The results from the simplified model, modified model and modified model with twice the abdominal area were compared. The time histories for lateral–directional states are shown in Figure 14.

### 6.3. Discussion of Manoeuvring Using Abdominal Deflection

Figure 13 illustrates the effects of upward abdominal deflection on the longitudinal states of a dragonfly-inspired aircraft model during a manoeuvre. The time history shows the performance of three different models: the simplified model (considering only inertial effects), a modified model including aerodynamic effects, and another modified model with twice the nominal abdominal area.

The results indicate that the simplified model, which only accounts for inertial effects, exhibits the highest manoeuvre effectiveness, as demonstrated by the maximum average acceleration and absolute pitch rate after 3 s. When aerodynamic effects are included, the effectiveness of the manoeuvre decreases due to the additional drag introduced by the abdomen. Considering the percentage average variations summarised in Table 4, the inclusion of aerodynamic effects reduced manoeuvre effectiveness in terms of acceleration and pitch rate by about 0.06% and 30.26%, respectively. The effectiveness was further reduced by about 0.11% and 56.70%, respectively, when the abdominal area was doubled, highlighting the significant impact of increased drag.

The graphs in Figure 13 also suggest that increasing the abdominal area reduces manoeuvre effectiveness even more. The larger abdominal area introduces more aerodynamic drag, which diminishes both acceleration and pitch rate during the manoeuvre. Overall, these results show the importance of considering both inertial and aerodynamic contributions when designing and simulating insect-inspired flight systems. While abdominal deflection can effectively initiate manoeuvres, the aerodynamic effects and the size of the abdomen play a subsequent role in determining the overall effectiveness of these manoeuvres.

Although the roll, pitch, and yaw rates presented in Figure 14 are quite oscillatory over time, useful conclusions can still be drawn. The aerodynamic effects of the abdomen, as shown in Figure 14, increase the magnitude of drag-dependent steering manoeuvres and introduce a non-transient torque input, unlike the inertial effects that cease once the abdomen stops accelerating or decelerating. The combination of inertial effects, which remain consistent regardless of drag area, and drag-based steering becomes increasingly effective at producing torques and influencing the aircraft’s dynamics, especially as the abdominal area increases.

## 7. Discussion

We have demonstrated the mathematical framework by exploring the behaviour of an example aircraft that was not specifically modelling a particular insect, although it drew some inspiration from dragonflies. The formulation we have presented provides a tool to explore the space of appendage movement, revealing the underlying effects that come with the approach. To achieve a full system view, it would be necessary to implement expressions for the costs, limits and mass of the actuators required to move the appendage. Given what is known about insect flight and appendage motion, there is no doubt that there is some value in the mechanism, but some important considerations are related to the specifics of insects. For example, they must have abdomens, since they contain the necessary parts of the digestive system. Actuating a part that is necessary is quite a different systems engineering consideration from adding a part in order that it be actuated. This implies that careful consideration of value is needed, or alternately, that a use would be found for an appendage behind the likely centre of gravity. Some aircraft configurations might have distinctly different appendages than the simple case here, and have more of them simultaneously. For example, some flying insects have large antennae, long legs, horns, cerci and so on. It would seem that different observations would be made from a leg-dominated flying insect, which would have the potential to use its legs to create very high drag, but with little inertia.

Considering the outcomes for this aircraft, and not considering some constraints such as actuator mass, redundancy and so on, the drag added to the model more realistically captures the cost of deflecting appendages, leading to generally worse performance for lateral and longitudinal manoeuvres than an inertial appendage alone. This is reasonable, as drag is a cost for the deflection, and the wings will generally provide less costly manoeuvres, until they run out of authority, in which case the abdomen can add more authority. It should be noted that there are cases in aerial combat where losing energy rapidly may be useful, and abdominal drag may provide value.

This paper simplified some conditions that need to be considered in the future. To address the limitations, the current study could benefit from a more sophisticated aerodynamic model that accounts for the interaction between the wings and abdomen, as well as the unsteady aerodynamic forces that arise during rapid abdominal deflection. The simplified model used in this study assumes negligible aerodynamic interference and relies on a quasi-steady approach, which may not fully capture the complexity of real-world flight dynamics, particularly during dynamic manoeuvres. Future research could incorporate Computational Fluid Dynamics simulations or experimental wind tunnel studies to better quantify these interactions and refine the model.

Additionally, while the current results provide valuable insights, they would be strengthened by experimental validation through wind tunnel tests or flight experiments with physical prototypes. This would help to verify the theoretical predictions and ensure that the model accurately represents the behaviour of insect-inspired UAVs under various flight conditions. Addressing these areas in future work would enhance the robustness and applicability of the findings, contributing to the development of more advanced and manoeuvrable UAV designs.

## 8. Conclusions

This paper introduced a tensor formulation of aircraft flight dynamics that included both the aerodynamics and dynamics of appendages in addition to wings. We showed that the framework allowed the modelling and exploration of the effects of surface area on the dynamic/inertial and aerodynamic contributions of a deflectable appendage, and how these contributions affect the forward flight performance and manoeuvrability of a bio-inspired aircraft. Considering a cylindrical abdomen as the deflectable appendage, results showed that the aerodynamic contribution of the insect-inspired abdominal appendage (mainly drag) increased with increasing area, thereby reducing performance due to the increased power and energy required in steady cruise flight. However, the initiation of a steady pull up manoeuvre from trim using the abdominal appendage instead of the elevator provides a higher manoeuvre advantage despite drag. The results also showed that it is indeed possible for the abdominal appendage to initiate dives and turns; however, the manoeuvre effectiveness depends on the shape. For the dive manoeuvre, the effectiveness worsened with increased size, whereas, for turning, the effectiveness improved with increased size. The framework developed here will allow a consideration of appendages in the conceptual design of novel biomimetic airframes and control systems. Future work will consider models of energy, weight and power of actuators that would be needed to implement this means of flight control in such a way as to provide an advantage to autonomous aircraft.

## Figures and Tables

**Table 1:** Aircraft model’s physical properties [41].

Parameter	Value	Parameter	Value
$m^{B}\;\left(\mathrm{kg}\right)$	0.325	$m^{T}\;\left(\mathrm{kg}\right)$	0.06
Body length, $l_{B}\;\left(m\right)$	0.3	Tail length, $l_{T_{max}}\;\left(m\right)$	0.4
Max. body diameter, $d_{B_{max}}\;\left(m\right)$	0.14	Tail diameter, $d_{T}\;\left(m\right)$	0.05
${I_{xx}}_{b}^{B}\;\left(\mathrm{kg}\;\cdot \;m^{2}\right)$	0.00187	$b_{ref}\;\left(m\right)$	1.4
${I_{yy}}_{b}^{B}\;\left(\mathrm{kg}\;\cdot \;m^{2}\right)$	0.01117	$c_{ref}\;\left(m\right)$	0.19434
${I_{zz}}_{b}^{B}\;\left(\mathrm{kg}\;\cdot \;m^{2}\right)$	0.00934	$S_{ref}\;\left(m^{2}\right)$	0.26865
$cg_{b}^{B}\;\left(m\right)$	[−0.064; 0; 0.003]	ARP(m)	[0.025; 0; 0]

**Table 2:** Percentage average variation in steady cruise flight performance parameters compared to the simplified/inertial model.

**V (m/s)**	**Percentage Average Variation (%)**			
	**Power** **Required**		**Elevator** **Deflection**	
5	0.7667	1.5317	0.1656	0.3290
10	3.0853	6.1339	0.5732	1.2012
15	3.6385	7.2757	−3.2011	−6.9674

**Table 3:** Energy manoeuvrability comparison of average specific energy and average specific excess power using $\Delta \delta_{e}$ or $\Delta \theta_{T}$.

Control Effector and Model Used	Average Specific Energy (m)	Average Specific Excess Power (m/s)
$\Delta \delta_{e}$, Inertial	105.093	−0.014
$\Delta \theta_{T}$, Inertial	105.103	0.0043
$\Delta \delta_{e}$, Inertial + Aero	105.0927	−0.0138
$\Delta \theta_{T}$, Inertial + Aero	105.1015	0.0042

**Table 4:** Percentage average variation compared to the simplified model with nominal abdominal area.

**Modified Model**	**Percentage Average Variation (%)**		
	**Velocity**	**Height**	**Pitch Rate**
Area × 1	$-5.5190\times 10^{-2}$	$6.4369\times 10^{-3}$	$-3.0245\times 10^{1}$
Area × 2	$-1.0675\times 10^{-1}$	$1.2404\times 10^{-2}$	$-5.6702\times 10^{1}$

## Data Availability

Data are contained within the article.

## Nomenclature

${\left[*\right]}^{A}$	* (model, vector or tensor) expressed in arbitrary reference frame A (Inertial (I), central body (B) and abdominal (T) reference frames in text)
*A*	Arbitrary Rigid body A (Central body (B), abdomen (T) or whole aircraft (C) in text)
${\left[R\right]}^{BA}$	The rotation matrix from reference frame A to B
b, t, j, c	Locations of the centre of mass of the central body, tail, tail joint and whole aircraft, respectively
$s_{ab},\;S_{ab}$	Displacement, skew-symmetric matrix of displacement of point *a* relative to point *b* (*m*)
$V_{A}^{B}\;$	Velocity of point A relative to point B (m/s)
$m^{A},\;m$	Mass of rigid body A, total mass of the aircraft, respectively (kg)
$I_{a}^{A}$	Inertia tensor of rigid body *A* about point *a* (${\mathrm{kg}\;m}^{2}$)
$\omega^{AB},\Omega^{AB}$	Angular velocity, skew symmetric matrix of angular velocity of frame A relative to frame B, respectively (rad/s)
*F*	External force vector (*N*)
*M*	External moment vector (*N m*)
**Superscripts**	
˙	First-order time derivative
¨	Second-order time derivative

## Appendix A. Athena Vortex Lattice File for Aerodynamic Data

Below is the AVL input geometry file used to generate the aerodynamic data of the dragonfly-inspired straight-wing aircraft used in this study. The aerodynamic coefficients and stability derivatives are functions of the aerodynamic angles. To generate the data as a function of the angle of attack, for instance, AVL is capable of sweeping through a range of AoA. More information on how to use the AVL tool can be found in [50].

## Appendix B. Model Validation: Single-Body vs. Multibody

The verification/validation of the proposed multibody model is presented in this section. The states and control inputs for the aircraft considered are as follows:

(A1) $$x={\left(u,v,w,p,q,r,\phi ,\theta ,\psi ,X,Y,Z\right)}^{T},$$

(A2) $$u={\left(\delta_{e},\delta_{a},T_{n},\phi_{T},\theta_{T},\psi_{T}\right)}^{T},$$

The single-body and multibody model of the same aircraft should present the same longitudinal trim results. The translational and rotational flight dynamics equations of motion for a single rigid body not referenced to the centre of mass have been derived by Bacon and Gregory in [58] as

(A3) $$\begin{array}{cc} \; & m\;\left({\left[{\dot{V}}_{b}^{I}\right]}^{B}+{\left[\Omega^{BI}\right]}^{B}{\left[V_{b}^{I}\right]}^{B}\right)+\left({\left[{\dot{\Omega }}^{BI}\right]}^{B}m{\left[s_{cb}\right]}^{B}\right)\\ \; & +\left({\left[\Omega^{BI}\right]}^{B}\;\left({\left[\Omega^{BI}\right]}^{B}m{\left[s_{cb}\right]}^{B}\right)\right)={\left[F\right]}^{B} \end{array}$$

(A4)
$$\begin{array}{cc} \; & \left(m{\left[S_{cb}\right]}^{B}{\left[{\dot{V}}_{b}^{I}\right]}^{B}\right)+\left(m{\left[\Omega^{BI}\right]}^{B}\left({\left[S_{cb}\right]}^{B}{\left[V_{b}^{I}\right]}^{B}\right)\right)\\ \; & \left(m{\left[V_{b}^{I}\right]}^{B}\left({\left[\Omega^{BI}\right]}^{B}{\left[S_{cb}\right]}^{B}\right)\right)+{\left[I_{b}^{C}\right]}^{B}{\left[{\dot{\omega }}^{BI}\right]}^{B}\\ \; & +\left({\left[\Omega^{BI}\right]}^{B}{\left[I_{b}^{C}\right]}^{B}{\left[\omega^{BI}\right]}^{B}\right)={\left[M\right]}^{B}+\left[{\left[S_{cb}\right]}^{B}{\left[F\right]}^{B}\right] \end{array}$$

The longitudinal trim results of the single-body model in [58] and the multibody model presented in this paper are calculated and compared. For the single-body model, the aircraft rotation of the abdomen was not considered during the estimation for longitudinal trim. The longitudinal trim using the single-body model can be described as follows: for a given airspeed *V* and a fixed abdominal pitch angle $\theta_{T}$, the objective is to estimate the appropriate longitudinal pitch angle θ and control, elevator $\delta_{e}$ and thrust $T_{n}$ to make the longitudinal rates, $\dot{u},\dot{w},\dot{q}$ and $\dot{h}$, approach zero.

In the case of the multibody model, the longitudinal trim can be described as follows: for a given airspeed *V* and a fixed abdominal pitch angle $\theta_{T}$, the objective is to estimate the appropriate longitudinal pitch angle θ and controls, elevator $\delta_{e}$ and thrust $T_{n}$ required to keep the abdomen pitch angle $\theta_{T}$ at a given value to make the longitudinal rates $\dot{u},\dot{w},\dot{q},\dot{h}$ and ${\left[{\dot{\omega }}^{TB}\right]}^{T}$ approach zero. Because the abdominal motion dynamics are included in the multibody model, the torque $\tau_{ay}$ required to keep the abdominal pitch inclination angle at the specified value can also be estimated.

The longitudinal trim problem is solved in MATLAB/Simulink for an airspeed $V_{0}=10$ m/s, a height $H_{0}=100$ m and abdominal pitch inclination angles $\theta_{T_{0}}=0,-10$ and −30°. The results are presented in Table A1.

**Table A1:** Single-body vs. multibody trim results at steady cruise trim condition.

$V_{0}$ (m/s)	$H_{0}$ (m)	$\theta_{T_{0}}$ (°)	Model Type	$\theta_{0}$ (°)	$\delta_{e0}$ (°)	$T_{n_{0}}$ (N)	$\tau_{\mathrm{ay}0}$ (Nm)
10	100	0	Single-body	−11.35	0.228	0.679	-
			Multibody	−11.35	0.228	0.679	−0.235
		−10	Single-body	−10.66	0.0707	0.683	-
			Multibody	−10.66	0.0707	0.683	−0.232
		−30	Single-body	−6.02	−0.96	0.708	-
			Multibody	−6.02	−0.96	0.708	−0.204

The trim results shown in Table A1 for the aircraft using the single-body model and multibody model exhibit similarities across different flight speeds. The key difference lies in the motor torque $\tau_{ay}$ for the abdomen, which represents the torque required to keep the abdominal pitch inclination angle at the specified values and can be considered a disturbance torque for control purposes.
